# Recent Advances in Nanostructured Transition Metal Carbide- and Nitride-Based Cathode Electrocatalysts for Li–O_2_ Batteries (LOBs): A Brief Review

**DOI:** 10.3390/nano10112106

**Published:** 2020-10-23

**Authors:** K. Karuppasamy, K. Prasanna, Vasanth Rajendiran Jothi, Dhanasekaran Vikraman, Sajjad Hussain, Jung-Hoon Hwang, Hyun-Seok Kim

**Affiliations:** 1 Division of Electronics and Electrical Engineering, Dongguk University-Seoul, Seoul 04620, Korea; karuppasamyiitb@gmail.com (K.K.); v.j.dhanasekaran@gmail.com (D.V.); junghoonh0326@gmail.com (J.-H.H.); 2 Avesta Battery & Energy Engineering, Ransbeekstraat, 310, 1120 Brussels, Belgium; pras.chemical@gmail.com; 3 Department of Chemical Engineering, Hanyang University, Seoul 04763, Korea; vasanthrj7878@gmail.com; 4 Graphene Research Institute, Sejong University, Seoul 05006, Korea; shussainawan@gmail.com; 5 Institute of Nano and Advanced Materials Engineering, Sejong University, Seoul 05006, Korea

**Keywords:** Li–O_2_ battery, molybdenum carbide, titanium carbide, nitrides, OER/ORR, Li_2_O_2_

## Abstract

A large volume of research on lithium–oxygen (Li–O_2_) batteries (LOBs) has been conducted in the recent decades, inspired by their high energy density and power density. However, these future generation energy-storage devices are still subject to technical limitations, including a squat round-trip efficiency and a deprived rate-capability, due to the slow-moving electrochemical kinetics of both the oxygen evolution reaction (OER) and oxygen reduction reaction (ORR) over the surface of the cathode catalyst. Because the electrochemistry of LOBs is rather complex, only a limited range of cathode catalysts has been employed in the past. To understand the catalytic mechanisms involved and improve overall cell performance, the development of new cathode electrocatalysts with enhanced round-trip efficiency is extremely important. In this context, transition metal carbides and nitrides (TMCs and TMNs, respectively) have been explored as potential catalysts to overcome the slow kinetics of electrochemical reactions. To provide an accessible and up-to-date summary for the research community, the present paper reviews the recent advancements of TMCs and TMNs and its applications as active electrocatalysts for LOBs. In particular, significant studies on the rational design of catalysts and the properties of TMC/TMN in LOBs are discussed, and the prospects and challenges facing the continued development of TMC/TMN electrocatalysts and strategies for attaining higher OER/ORR activity in LOBs are presented.

## 1. Introduction

The environmental pollution and climate change caused by the use of fossil fuels have been major causes of concern worldwide. To restrict the consumption of fossil fuels, alternative renewable energy storage devices with an enhanced energy density and power densities, including lithium-ion batteries (LIBs) [1,2,3,4], lithium–air (Li–O_2_) batteries (LOBs) [5,6], and supercapacitors (SCs) [2,7,8], have been developed for use in applications such as hybrid electric vehicles, off-grid electricity, and miniaturized electronic devices. LIBs and LOBs are also widely employed in high-energy devices due to their longer self-discharging time and excellent specific capacity. The specific energy density of LOBs (a theoretical and practical energy density of 5200 Wh kg^−1^ and ~1000 Wh kg^−1^, correspondingly) is larger than that of LIBs (200 Wh kg^−1^) [9], meaning that LOBs are able to power electric vehicles for more than 500 km on a single charge. 

However, their poor capacity, large polarization during the charge–discharge process, short lifespan, sudden fade in capacity, and sluggish electrochemical kinetics for the oxygen evolution reaction (OER) and the oxygen reduction reaction (ORR) has restricted commercialization of LOBs, while their storage capacity also needs to be increased for commercialization. In addition, the large-scale production of these batteries needs further research attention because it is not easy to attain high energy and power density simultaneously and because current fabrication processes are expensive and have safety issues. To overcome these drawbacks, nano-carbonaceous materials such as graphene, graphene oxide, carbon nanotubes (CNTs) and, their composites, transition metal chalcogenides, metal–organic frameworks (MOFs), and transition metal carbides (TMCs) and nitrides (TMNs) and their two-dimensional (2D) layered composites (MXenes) have been investigated because of their inherent synergistic physicochemical and electrochemical properties.

In the past two decades, the discovery of graphene and its unique properties has opened up a new avenue of research with regards to 2D materials [10,11,12,13,14,15]. Graphene and its composites have thus been broadly utilized in energy storage and conversion devices, but, due to the mono-layer thickness of graphite, they offer only a limited range of applications. On the other hand, graphene analogues, including 2D layered transition metal oxides, phosphides, sulfides, and selenides, have been considered capable candidates for use in energy storage devices because of their ultrathin nature and planar topology [16]. However, due to their low working potential, poor specific capacity, weak cyclability, and high-temperature synthesis, these compounds have yet to be commercialized. In the past decade, MOFs and their nanocomposites have also been investigated as excellent candidates for use in LIBs and LOBs owing to their adjustable physicochemical characteristics and ultra-high surface area. Nevertheless, the cycling performance of MOF-based electrodes is inferior to transition metal sulfides and selenides [17].

**Figure 1:**
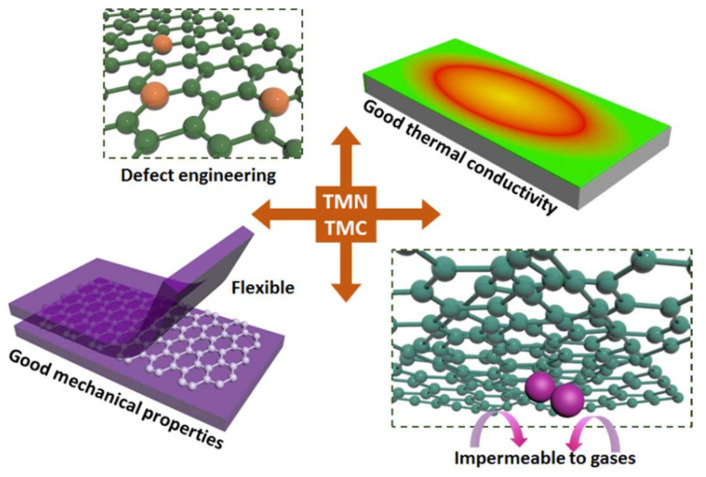
Schematic illustration of the properties of transition metal carbides (TMC)/ transition metal nitrides (TMN) including MXenes.

In recent years, numerous efforts have been made to find an alternative to noble metal catalysts and to design non-precious metal catalysts, including 2D transition metal chalcogenides, metal oxides, and metal carbides and nitrides. Of these, TMCs and TMNs have received particular attention due to their outstanding catalytic performance and high electrode material stability, which is similar to that exhibited by commercial Pt-C catalysts. In addition, their 2D layered form MXene and its composites have been employed in various materials science applications, including energy storage devices. As shown in Figure 1, TMC/TMNs including MXenes offer high electrical conductivity, hydrophobicity, the quick diffusion of ions and molecules, easily adjustable structures, good thermal stability, a tunable structure and thickness, and a high surface area [18,19].

To date, a handful of reviews have been published on energy storage and conversion applications involving metal carbide and nitride-based nanostructured electrodes [20,21]. In particular, a number of reviews have summarized the synthetic strategies for and the role of MXenes in energy storage and conversion based on studies published before 2017 [22,23,24,25]. Very recently, Jun et al. provided a complete overview of MXene compounds for environmental and electrochemical storage applications [26], while the mechanisms of delamination and the role of MXene electrodes in dictating the cycling properties of LIBs were examined thoroughly by Zhang et al. [27]. However, these studies have not addressed the evolution of TMCs/TMNs into 2D layered TMCs/TMNs (i.e., MXene-based compounds) in the context of LOBs, and the electrochemical performance and storage properties of these batteries have not been summarized in detail. Therefore, a review that specifically focuses on nanostructured TMC/TMN cathode catalysts for LOBs has been long overdue. Because the role of anodes and electrolytes in LOBs have been extensively reported elsewhere [28,29], we do not address these in the present review; rather, we provide a comprehensive review of the electrochemical, physicochemical, and storage properties of both bulk and 2D layered TMCs and TMNs, counting the carbides and nitrides of Ti, V, Fe, and Mo and their composite counterparts, when used as active cathode catalysts in rechargeable LOBs. 

## 2. Basic Principles of Li–O_2_ Batteries

**Figure 2:**
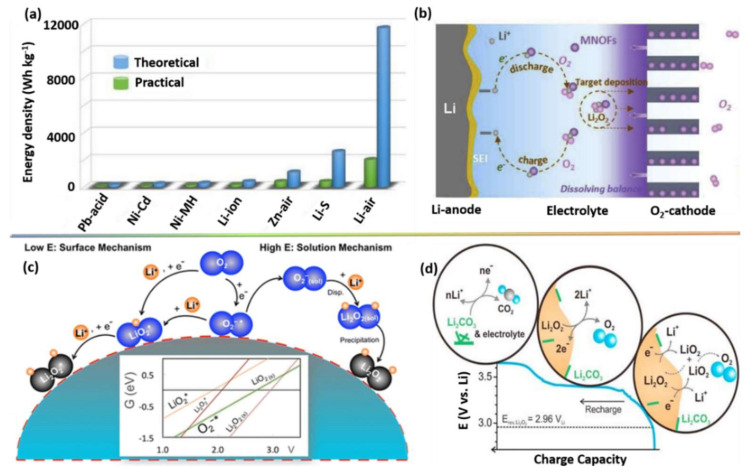
(**a**) Theoretical and practical energy density of various energy storage devices [30]. Copyright 2020 Elsevier. (**b**) Graphic illustration of different components of an Li–O_2_ battery (LOB) [6]. Copyright 2020 Elsevier. (**c**) Expected oxygen reduction reaction (ORR) mechanisms in an LOB [39]. Copyright 2016 The American Chemical Society. (**d**) Expected oxygen evolution reaction (OER) mechanisms in an LOB [40]. Copyright 2013 The American Chemical Society.

LOBs represent a rechargeable energy storage technology that has received significant research attention as a way to meet future energy demands. In theory, LOBs have a higher energy density than gasoline and other storage devices [30] (Figure 2a), thus, they are expected to replace gasoline for use in road, aquatic, and aerial vehicles [31,32,33,34,35,36,37,38,39,40]. 

Littauer et al. first described the interesting Li–O_2_ chemistry in 1976 [35], after which Abraham et al. demonstrated the LOB system using a non-aqueous electrolyte in 1996 [36]. As shown in Figure 2b, a typical LOB system consists of a negative electrode (Li metal), a positive electrode (primarily a carbonaceous material), and an electrolyte (generally divided into aqueous, non-aqueous, hybrid, and solid electrolytes) [6]. During the electrochemical reaction in LOBs, Li_2_O_2_ is formed as a by-product of the interaction between O_2_ and Li^+^ during the discharge process before decomposing again during subsequent charging.

Because the positive electrode (i.e., cathode) provides a reaction site for the electrochemically active O_2_ and Li^+^ and stores Li_2_O_2_, it plays pivotal role in the storage capability of LOBs and the reversibility of Li-ions [37,38]. A significant volume of research activity has thus been devoted to the optimization of cathode electrodes in LOBs. The specific surface area, electrical conductivity, pore size, and volume of the cathode active material are considered the most influential properties for enhancing the storage capability and reversibility of LOBs. The electrochemistry of cathode electrodes needs to be well-understood in order to identify optimum active cathode materials for use in LOBs. At the cathode electrode, Li_2_O_2_ is formed via the ORR in two steps: (1) O_2_ binds with unoccupied O-positions to produce O_2_^−^ (superoxide) and (2) O_2_^−^ reacts with Li through the dismutase process to produce Li_2_O_2_. A schematic illustration of the ORR in a LOB is presented in Figure 2c [39]. Because the decomposition voltage of Li_2_O_2_ is much higher (3.75 V vs. Li/Li^+^) than the plateau potential of 3.4 V, it is unstable. As illustrated in Figure 2d, the decomposition of Li_2_O_2_ occurs via the OER in another two-step process [40]: (1) Li_2_O_2_ releases one electron and decomposes to LiO_2_ and (2) LiO_2_ interacts with oxygen vacancies to release the second electron and lithium ions. As a result, LOB operation completely relies on the OER and ORR during the electrochemical charge–discharge process [41,42,43]. 

### 2.1. Basic Requirements for an Air Cathode

The chemical and physical properties of Li_2_O_2_ determines the capacity, cycle overpotential, rate capability, and cycle life. Cathode electrodes in LOBs are thus designed in consideration of the physical and chemical properties of Li_2_O_2_. In general, the choice of an air electrode for a rechargeable LOB depends on maximizing its Li_2_O_2_ ability with stronger ORR and OER electrocatalytic behavior. In particular, stable air cathodes should have the following properties to achieve better LOB performance:

- High electrochemical and chemical stability are necessary to reduce the overpotential during charging, which results in a reversible electrochemical reaction at satisfactory charge–discharge potentials, with less chance of irreversible sponging reactions.
- A high specific surface area with a mesoporous structure enhances the discharge capacity even at high current densities, which is an important criterion for the high storage of Li_2_O_2_.
- To increase the rechargeability, a large packed electrode with a lower void volume is essential because it can prevent electrolyte penetration and improve the electrochemical reaction at catalytic sites due to the enhanced transportation of active O_2_ and Li^+^. Therefore, an electrode with a porosity that is appropriate for the size of Li_2_O_2_ exhibits greater rechargeability.
- Electrical conductivity is a deciding factor for renewable energy storage performance; hence, a cathode with greater electrical conductivity is required to allow the consistent transportation of electrons from the insulator and Li_2_O_2_ to the surface of the cathode.

Based on these considerations, various nanostructured carbonaceous materials have been investigated as active cathode materials in LOBs [44,45]. Carbonaceous materials are predominantly used as a cathode in LOBs due to their high specific surface area for the formation of Li_2_O_2_. To enhance the properties of these carbon materials, their morphology can be changed into CNTs and carbon nanofibers (CNFs). The one-dimensional (1D) architecture of CNTs and CNFs enhances the efficiency of charge transfer and the dimensional stability of the electrode during the charge–discharge process and its cycle life [46,47]. Despite their advantages, CNTs and CNFs have issues with the agglomeration of particles during the electrochemical process, which weakens the performance of LOBs. To overcome this, 2D graphene was introduced as potential cathode active material for LOBs, though the re-stacking of graphene sheets as the number of cycles increases has become a concern [48,49,50]. To avoid this re-stacking, various metals, metal oxides, and heteroatoms have been introduced into the graphene architecture [51,52,53]. The synthesis of three-dimensionally (3D) ordered mesoporous carbon and MOFs have been introduced to avoid the self-aggregation of carbon particles [54]. Not only does the aggregation of carbon particles cause concern, the formation of Li_2_CO_3_ during the charge–discharge process is also a problem. The emergence of an insulating layer Li_2_CO_3_ on the exterior layer of the carbon electrode increases the interfacial potential, resulting in higher charging potentials and a lower round-trip efficiency for LOBs [55,56].

Recently, carbon-based cathode electrodes have been replaced with other materials based on metals, metal oxides, carbides, nitrides, sulfides, and their composites to prevent the formation of Li_2_CO_3_ as a by-product. After extensive research, nanoporous gold and platinum have been identified as the most promising metals for the decomposition of Li_2_O_2_ at a low potential, but the cost of electrode fabrication is a major concern [57]. In terms of oxide-based materials, V_2_O_5_ [58], Co_3_O_4_ [59], Al_2_O_3_ [60], and MnO_2_ [61] have generated significant interest, but these materials induce a significant increase in potential that acts as a hurdle for the transfer of electrons. Currently, carbide- and nitride-based cathode electrodes are under investigation as materials for energy storage devices [62,63]. 

### 2.2. Characterization Techniques for TMCs and TMNs Cathode Catalysts

As we mentioned above, the present review is primly focused on the cathode catalysts for LOB applications. Some of the significant physicochemical and electrochemical characterization techniques for TMCs and TMNs are discussed in this section.

#### 2.2.1. Physicochemical Characterization Techniques

In general, the anode or cathode materials are characterized physicochemically through various type of spectroscopy and microscopy techniques such as field emission-scanning electron microscopy (FE-SEM), high resolution transmission electron microscopy (HR-TEM), X-ray diffraction (XRD), and X-ray photoelectron spectroscopy (XPS) analyses. The microscopic analyses using FE-SEM and HR-TEM are used to probe the morphological evolution of electrode materials during the galvanostatic charge-discharge (GCD) process, which affords a vision into the favored reaction sites. The damage caused by the electron-beam of FE-SEM analysis is almost less than that caused by HR-TEM, which makes it feasible to analyze the reaction products and by-products such as Li_2_O_2_ and Li_2_CO_3_, respectively, which are highly unstable when exposed to an electron beam [64,65]. In order to avoid such risk factors, in-situ HR-TEM has employed to study the electrochemical process of Li–O_2_, which provides temporal and spatial resolution and does not mimic the cell’s environment as much as compared to other microscopic analyses. XRD study is used to determine and quantify the crystallinity of the reaction product/by-product include Li_2_O_2_ and Li_2_CO_3_. Furthermore, the electrochemical oxidation of the reaction site products can be monitored by in-situ operando-XRD analysis during GCD process. Another important technique of XPS has been used to evaluate the surface chemistry and spatial distribution of GCD reaction products, including Li_2_O_2_ and Li_2_CO_3_, and also provides the basic knowledge on these reaction products interactions with the electrolyte and electrodes. The BET analysis provides the complete understanding of porosity properties of the cathode catalysts such as pore volume, surface, and diameter and plays a key role to evaluate the discharge capacity of the LOB cell.

#### 2.2.2. Electrochemical Characterizations

Usually, the cathode catalysts for electrochemical tests are prepared in a standard procedure as described earlier [66]. In brief, the as-prepared TMCs/TMNs cathodes (loading mass = 1–3 mg) are mixed together with binders (Kynar^®^ HSV 900 and Kynar^®^ HSV 1810) and super carbon in a stoichiometric ratio of 3:1:1 or 4:1:1 in N-methyl-N’-pyyrolidone solvent to form a slurry. The attained slurry was coated over a substrate by doctor blade method. The thickness of the coating material is maintained around 90–100 μm. The coated substrates were dried under vacuum at 80–100 °C for about 12 h and then laminated into disks. The binder-free cathodes have also been studied and reported elsewhere owing to the binder’s decomposition with ether-electrolytes during the cycling process. The LOB assembly could be achieved in the following manner: a Swagelok cell consists of a piece of lithium foil over a stainless steel at the anode end and clean O_2_ is applied on as-prepared catalyst at the cathode end. The electrolyte either protic or aprotic immersed Celgrard/glass fiber separator is placed in between them; then, the whole cell set-up is sealed in a glass container that is filled with clean O_2_. Considering the sensitivity of LOBs, the whole experiment should be done under inert atmosphere. The prepared Swagelok cells can be subjected into multiple electrochemical characterization analyses such as cyclic voltammetry (CV), electrochemical impedance analysis (EIS), and galvanostatic charge-discharge (GCD) analyses. To identify the electrocatalytic mechanism and performance of LOB, CV, and GCD, analyses need to be performed at different scan rates and current rates, respectively. However, the formation of by-products such as Li_2_CO_3_ and LiOH during cycling process are unavoidable due to the side reactions, which include electrolyte and binder decomposition at the electrode and electrolyte interfaces. Nevertheless, the side reactions and formation of by-products can be largely controlled nowadays by the rational design of electrode materials and various synthesis strategies.

## 3. Metal Carbides for LOBs

### 3.1. Titanium Carbide

It is generally agreed that carbon cathodes are unstable in air, which is a major problem that hinders the commercialization of LOBs. This is because the functioning of an LOB strongly depends on the reversible formation and decomposition of Li_2_O_2_ over the surface of cathode. Hence, a stable cathode is essential to obtain better LOB performance. In this regard, titanium carbide (TiC)-based cathodes have been developed as a suitable alternative in recent years. TiC-based compounds have been widely investigated as potential electrodes in supercapacitors and LIBs because of their excellent electrical conductivity of 3 × 10^7^ Scm^−1^ and their general lack of side reactions during the electrocatalytic process, which in turn facilitates the formation and decomposition of Li_2_O_2_. Inspired by these properties, TiC-based catalysts have been widely employed in LOBs. 

Ottakamthotiyl et al. investigated TiC, SiC, and TiN, which are lightweight and chemically inert [67]. They employed two electrolytes, 0.5 M LiClO_4_ in dimethyl sulphoxide (DMSO) and 0.5 M LiPF_6_ in tetraethyleneglycoldimethylether (TEGDME). Li–O_2_ cells fabricated with a TiC electrode were tested using both electrolytes, and the charge–discharge behavior was investigated at 1 mA cm^−2^. Polarization was perceived to increase as the cycle number increased with the TEGDME electrolyte, thus, the capacity retention of Li–O_2_ cells with TEGDME was observed to be lower than with DMSO. The surface of the TiC electrode was evaluated after several charge–discharge cycles using X-ray photoelectron spectroscopy (XPS). Similar tests were also performed on SiC and TiN. The surface of the TiC electrode after several cycles contained significant levels of TiO_2_ and TiOC. However, on the SiC and TiN electrodes, oxide layers were not observed. The absence of an oxide layer on the SiC electrode inhibited the charging process, and thus, lowered the capacity with each cycle. In the absence of a TiO_2_ layer, TiN had a discharge potential of ~2.3 V and a capacity of ~100 mAh g^−1^, which was lower than the discharge potential (~2.55 V) and capacity of ~360 mAh g^−1^. The poor activity of TiN was ascribed to its lower conductivity in comparison with TiC. This report claimed that TiC with a DMSO electrolyte could achieve an Li_2_O_2_ purity of >99.5 for each discharge and complete oxidation during charging, with a >98% capacity retention after 100 cycles, which was far better than the results obtained using nanoporous gold electrodes and carbon electrodes [67]. 

It has been reported that TiC is unstable on a nanoscale and is readily converted into TiO_2_ and TiOC via thermodynamic reactions with Li_2_O_2_ and O_2_, meaning that the catalytic process is strongly hindered by a TiO_2_-rich layer in LOBs. Yang et al. [68] studied the effect of this TiO_2_-rich layer on the TiC surface using first-principle calculation. They also investigated the catalytic performance of oxidized TiC by comparing the O_2_ evolution barrier for the OER and the charge potential using density of states (DOS) analysis. Based on DOS calculations, it was confirmed that Li_2_O_2_ was readily adsorbed onto the (100) surface of TiC. In a similar manner, Raz et al. evaluated the adsorption energies of Li_2_O_2_, NaO_2_, and Na_2_O_2_ on the (111) surface of TiC using density functional theory and discussed the feasibility of using the prepared materials as electrodes for LOB and sodium ion batteries [69]. 

**Figure 3:**
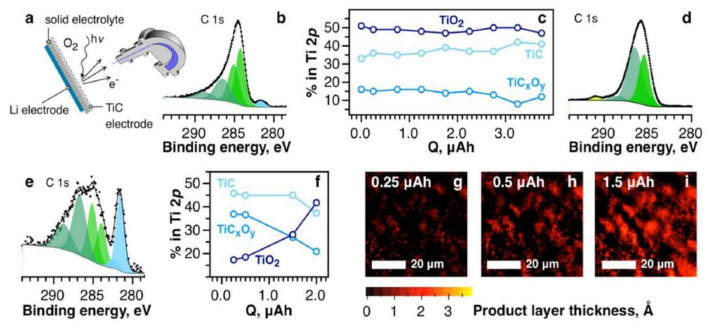
(**a**) Graphical representation of operando spectroscopy under an Ar atmosphere using an Li-metal, NASICON, and nanopowder TiC as anode, conducting the electrolyte and cathode, respectively. (**b**) C1s spectra of the TiC cathode before discharge (fresh cell). (**c**) Percentage of components in the Ti2p spectra during the discharge process (galvanostatic mode). (**d**) C1s spectra of the TiC cathode after discharge. (**e**) C1s spectra of the TiC cathode after Ar^+^ sputtering. (**f**) Percentage of components in the Ti2p spectra in a scrubbed electrode. (**g**–**i**) The discharge product layer’s effective thickness on an Ar^+^-sputtered TiC cathode at various discharge depths [70]. Copyright 2016 The American Chemical Society.

Following by Thotiyl et al., a detailed study on the deterioration of TiC and other Ti binary compound electrodes was conducted by Kozmenkova et al. They characterized the surface phenomenon of model chemical and electrochemical systems using in-situ and ex-situ experiments [70], in the process demonstrating the formation of a passivation layer during the ORR. A two-electrode cell system was fabricated by having TiC, lithium foil, porous Celgard separator immersed with 1 M lithium bis(trifluoromethane)sulfonylimide (LiTFSI) in a 1-ethyl-3-methy limidazoliumbis-(trifluoromethylsulfonyl)imide as cathode, anode, and electrolyte, respectively. The assembly was kept between two stainless steel (SS) plates, and the SS plate on the cathode side had a small hole to allow the in-situ analysis to be conducted. Figure 3a presents a schematic of the in-situ spectroscopic analysis of the solid-state lithium-conducting electrolyte built in an electrochemical cell. The interaction between the TiC and the ORR products and intermediates was analyzed in-situ to avoid the formation of by-products when in contact with liquid electrolytes. In the XPS chamber, the intensities of Li 1s and O 1s were observed to increase during galvanostatic discharge, indicating the advance of discharge products over surface layer of the cathode (Figure 3b–i) [70]. The surface of the TiC was stabilized by a protective coating layer of TiO_2_. Because TiO_2_ has a wide bandgap (3 eV), it places kinetic limitations on the electrons during the ORR. To overcome this limitation, the thickness of the cathode has to be optimized to offer both an acceptable electron transport rate and genuine surface protection. 

Adams et al. reported an interesting study on the significance of nanometric passivation on cathodes in LOBs. They tested TiC powders as received from two different suppliers (TiC-A and TiC-B) and compared them with TiN [71]. The as-received TiC powders were observed to have similar bulk properties but different surface properties. XPS and STEM analyses were carried out to observe the surface of the two TiC powders. TiC-A had an amorphous layer surrounding the particles and was around ~2–3 nm thick, whereas the amorphous layer was absent in TiC-B. The oxide layer on the TiC-A was predicted to be more than sufficient to completely prohibit electron transfer. The charge curves for the cathode TiO_2_, TiC-A, and TiC-B were compared with TiN. As predicted, the partially conducting TiO_2_, similar to TiN, did not support the OER due to the bulk insulating layer. Similarly, the TiC-A, with its amorphous layer, was inactive in terms of the OER. This is because the transfer of electrons through the insulating layer plays a critical role in LOBs; if the passivation layer is thin (e.g., TiC-B), sufficient Li_2_O_2_ could be charged at a fixed voltage, whereas if the layer is thick (e.g., TiC-A and TiN), the charging process is inhibited (Figure 3a) [71]. More research is required on the synthesis of active materials for cathodes to inhibit excessive oxidation and/or to form a conductive oxide layer on the material.

In addition to the oxidation of TiC cathodes via the thermodynamic interaction with Li_2_O_2_, another common limitation of these cathodes is their design. For instance, commercially available cathode powders with a binder mixture are unstable due to the decomposition of the binder. Thus, a balanced design for the ionic and electronic channels is necessary to transfer electrons to and from the Li_2_O_2_-insulated material. In this regard, 1D nanostructures such as nanowires and nanorods offer better catalytic and surface properties for cathodes, thereby improving the electrochemical kinetics of the catalytic reactions [72,73]. In addition, a 1D nanostructure is beneficial in terms of minimizing the grain boundary resistance, which in turn facilitates improved charge transportation over the cathode surface. 

**Figure 4:**
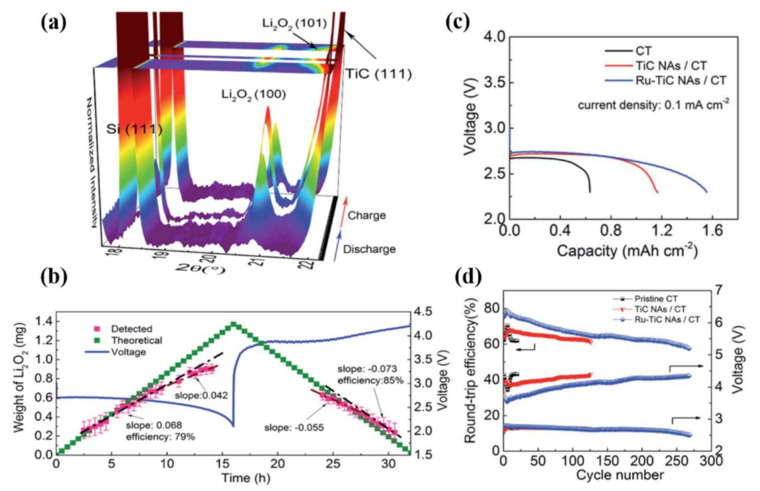
(**a**) Operando synchrotron radiation powder X-ray diffraction (SP-PXRD) patterns for a Ru-TiC NAs/CT electrode at a wavelength of 0.994 A, recorded every 30 min during the first cycle at 0.1 mA cm^−2^. (**b**) Corresponding galvanostatic charge–discharge process (first cycle) and mass evolution of the discharge product Li_2_O_2_ [74]. Copyright 2018 The Royal Society of Chemistry. (**c**) Galvanostatic discharge plot and (**d**) round-trip efficiency and half-capacity voltage for each cycle with Ru-TiC NAs/CT, TiC NAs/CT, and CT electrodes [74]. Copyright 2018 The Royal Society of Chemistry.

In this vein, Ru-TiC nanowire arrays developed in-situ on a carbon textile were employed as a potential cathode for rechargeable LOBs [74]. As-prepared free-standing cathodes with and without Ru-support were characterized for their use in LOBs. A TiC nanowire array (TiC NA/Ru) with Ru-support exhibited higher OER/ORR activity and excellent cycling stability due to the uniform TiC nanowires grown perpendicular to the carbon textile surface without the need for a binder. The length and width of the TiC nanowires on the carbon skeleton were 30–50 μm and 200–500 nm, respectively. The performance of an LOB with Ru-based TiC electrodes was characterized using synchrotron radiation powder XRD (SR-PXRD) during the charge–discharge process (Figure 4a,b). The TiC NA and TiC NA/Ru-based LOBs demonstrated an excellent capacitance of 352 mAh g^−1^ and 468 mAh g^−1^, respectively, at 29.3 mAg^−1^ (Figure 4c,d).

**Figure 5:**
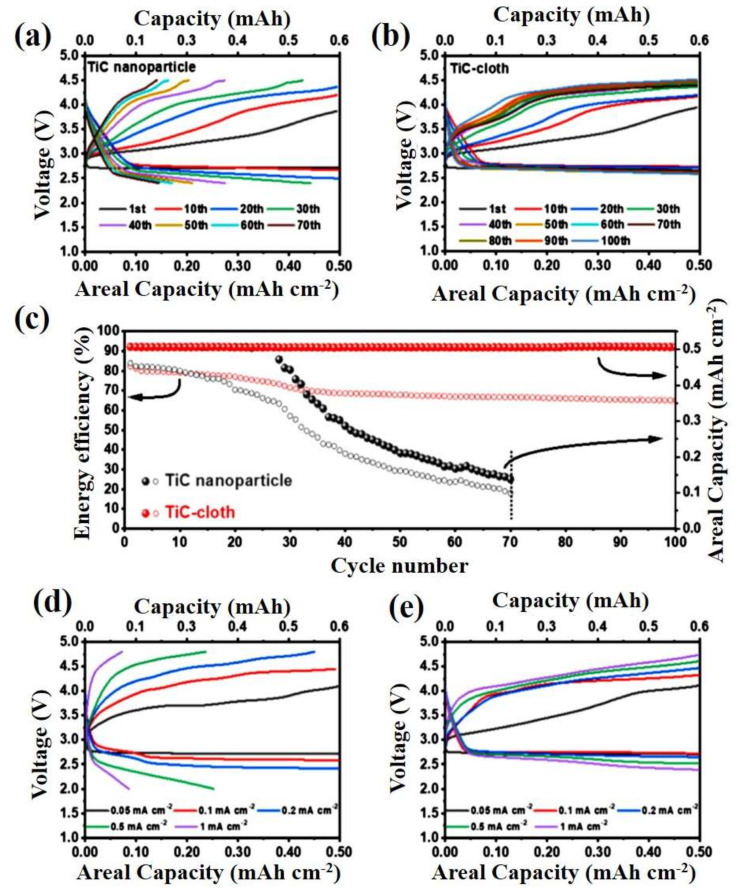
Galvanostatic charge-discharge (GCD) plot for an LOB using (**a**) a titanium carbide (TiC) nanoparticle (NP) cathode @ 0.1 mA cm^−2^ for a duration of 5 h and (**b**) a TiC-cloth cathode @ 0.1 mA cm^−2^ for a duration of 5 h. (**c**) Relationship between the areal capacity, energy efficiency, and number of cycles. (**d**) Discharge plot for various current densities with a TiC NP cathode. (**e**) Discharge plot for various current densities with a TiC-cloth cathode [75]. Copyright 2020 Elsevier.

Recently, binder-free, 3D-nanostructured TiC wire was grown perpendicular to a carbonized cotton T-shirt (acting as an air electrode) to develop self-standing electrodes for LOBs [75]. Due to the light weight of the substrate compared to commercially available carbon clothes, an outstandingly high energy density was produced. As shown in the discharge plateau in Figure 5a, the discharge profiles of the TiC wire on TiC cloth and pristine TiC nanoparticles (NPs) were the same for the initial cycles. However, the cathode TiC NPs ended with a discharge voltage of 2.4 V before attaining the measured capacity after 30 cycles. In contrast, the TiC wire on TiC cloth had a stable discharge profile even after 100 cycles with a discharge and charging voltage of 2.7 V and 4.3 V, respectively (Figure 5b,c). The TiC wire on TiC cloth also maintained a constant energy efficiency, while the other electrodes exhibited a sudden drop. Based on the rate performance plot in Figure 5d, the pristine TiC NP electrode failed to achieve the desired capacity at 0.2 mA cm^−2^ (Figure 5e) and showed exciting charge-discharge terminal potentials.

### 3.2. Iron Carbide

**Figure 6:**
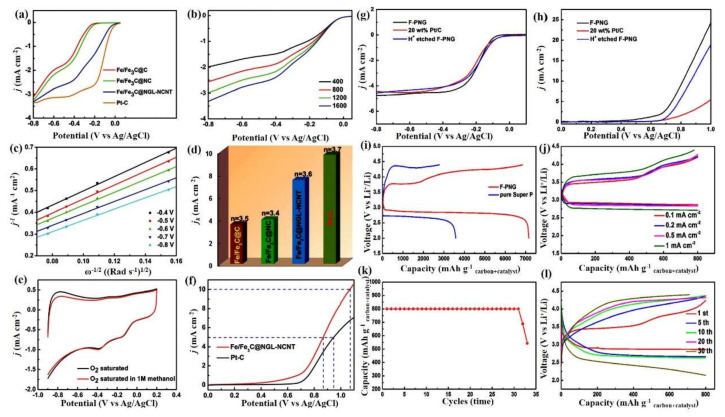
(**a**) Linear sweep voltammogram (LSV) for various Fe3C-Fe-based cathode catalysts at 1600 rpm. (**b**,**c**) LSV and related K-L curves for an Fe/Fe_3_C@NGL/NCNT electrode. (**d**) Plot of the kinetic limiting current density against electron transfer. (**e**) Cyclic voltammogram (CV) for an Fe/Fe_3_C@NGL/NCNT electrode in O_2_-saturated KOH and O_2_-saturated KOH/methanol solutions (both 0.1 M). (**f**) OER current density for Fe/Fe_3_C@NGL/NCNT and Pt/C electrodes in an O_2_-saturated 0.1 M KOH solution [80]. Copyright 2015 The Royal Society of Chemistry. (**g**) Comparison LSV for the ORR of H^+^-etched Fe-PNG and saleable Pt/C catalysts at 1600 rpm in a 0.1 M KOH solution saturated with O_2_ [83]. (**h**) Comparison LSV for the OER of H^+^-etched Fe-PNG and commercial Pt/C catalysts at 1600 rpm [83]. (**i**,**j**) GCD plot for Fe-PNG @ 0.1 mAcm^−2^ and various current density rates within a potential window of 2.0–4.4 V. (**k**) Cyclic behavior of an Fe-PNG electrode and (**l**) the corresponding GCD plot at 0.1 mA cm^−2^ in a voltage window of 2.0–4.4 V [83]. Copyright 2016 Elsevier.

In recent years, a large volume of electrocatalysis research has focused on replacing noble metals with non-precious metals such as transition metal oxides, chalcogenides, phosphides, carbides, and nitrides. In particular, abundantly available iron and iron carbide (Fe/Fe_3_C) have received significant attention in this respect due to their strong, inherent, and durable catalytic activity for the OER and ORR. In the early 2000s, Lefevre et al. [76] first reported an iron-based non-precious metal catalyst (Fe-NPMC) for polymer electrolyte fuel cells synthesized by twice ball milling a mixture of ferrous acetate and phenanthroline carbon support and then subjecting it to pyrolysis at 800 °C. They found that the current density of the iron-based cathode was almost equivalent to that of a Pt-based cathode at a cell potential of ≥0.9 V. Lee et al. [77] subsequently fabricated Fe/Fe_3_C on melamine foam, which exhibited excellent ORR behavior in an alkaline solution. In addition, a similar type of C-C supported Fe/Fe_3_C cathode demonstrated outstanding ORR behavior in neutral media [78]. Hu et al. investigated the ORR activity of Fe_3_C NPs embedded in graphitic layers in both alkaline and neutral media and confirmed their high catalytic activity and stability in both media [79]. A similar type of cathode, N-incorporated Fe/Fe_3_C/CNTs derived from an MOF, was reported by Li et al. [80], acting as a bifunctional catalyst for both the OER and ORR processes. As displayed in Figure 6a–f, the prepared hybrid catalyst exhibited enhanced ORR activity equivalent to that of Pt/C and high OER activity superior to that of Pt/C, making it a potential candidate for use in renewable energy technologies. Yang et al. [81] later synthesized N-doped graphitic layers with an Fe_3_C/Fe catalyst to improve the ORR activity and explored it as a potential alternative for Pt-C in Zn-air batteries and fuel cells. Recently, an MOF-derived Fe_3_C@N-CNT catalyst also showed promising ORR activity in alkaline media [82].

Based on the observations above, few reports on Fe_3_C/Fe-based cathodes have focused on LOBs. Lai et al. [83] prepared N-doped graphene@Fe/Fe_3_C (Fe-PNG) electrodes using a one-step carbonization process with an MIL-100 (Fe) MOF as an iron precursor. The prepared Fe-PNG cathode exhibited a porous 3D network morphology in which Fe_3_C/Fe NPs were evenly distributed on the outer layer of the graphene. This porous 3D nanostructure contained meso- and macro-pores that create space to accommodate the Li_2_O_2_ produced during discharge, thus being beneficial for the migration of Li^+^ and O_2_. The ORR and OER activity of the Fe-PNG electrode was monitored using linear sweep voltammetry analysis at 5 mVs^−1^ (Figure 6g,h). As can be seen in the voltammogram, the Fe-PNG cathode showed excellent OER and ORR stability compared to the pristine Fe_3_C/Fe cathode. Their corresponding charge–discharge behaviors are demonstrated in Figure 6i–l. The experiment was conducted in a potential window of 2.0 to 4.4 V (vs. Li/Li^+^), producing a charge and discharge capacity of 6850 and 7150 mAh g^−1^_carbon+catalyst_, respectively (Figure 6j). The voltage profile of an LOB using Fe-PNG offered an outstanding capacity retention of 800 mAhg^−1^_carbon+catalyst_ at 0.1 mAcm^−2^ (Figure 6k) and the cycling stability was maintained for over 30 cycles at different current densities (Figure 6l). The same research group analyzed a similar type of electrode, Fe/Fe_3_C@graphatic carbon embedded with CNTs (Fe@NG-NCNT) derived from Prussian blue, and studied its potential application as a potential cathode in LOBs [84]. The as-prepared Fe@NG-NCNT-based LOB delivered a maximum discharge capacity of 6966 mAh g^−1^ at 0.1 mA cm^−2^. 

Recently, a unique architecture—a CNF-doped Fe_3_C/Fe (Fe/Fe_3_C-CNF) cathode—was developed via an electrospinning process. Due to the combined effect between the CNFs and Fe_3_C/Fe, the catalytic activity was drastically enhanced, which in turn favored the enhancement of the capacity, cycling stability, and rate capability [85]. Wei et al. [86] also observed the synergistic effect of robust interactions between Fe_3_C and carbonitride, which illustrated the superior ORR activity and the moderate OER activity in a KOH solution compared to that for commercially available Pt-C electrodes. These observations undoubtedly pave the way for their potential use as a cathode in future-generation LOBs.

### 3.3. Metal Nitrides

#### 3.3.1. Titanium Nitride (TiN)

TMNs have been widely acknowledged for their outstanding physicochemical properties, such as their high corrosion resistance, robust hardness, and high thermal stability. In general, they are obtained by incorporating N heteroatoms in transition metal lattices, which exhibit the combined properties of ionic crystals, covalent solids, and metals [87,88,89]. According to Fischer’s molecular orbital diagram, carbon has a 2p^2^ electronic structure. Most of the transition elements, which are referred to as d-block elements in the modern periodic table, have five 3d4s electrons. Thus, there is no d-electronic structure in TiC, whereas it is present for VC. In a similar manner, the 2p^3^ electronic structure of the N-atom verifies the d-electronic structure of early TMNs; for instance, compared to scandium nitride (ScN-0-d electron), there is a d-electron in the electronic structure of TiN. As a result, metal carbides are one d-electron short compared to their corresponding nitrides, which has a major influence on their catalytic activity. However, the ORR catalytic activity of single early TMNs is comparatively lower than commercial Pt-C catalysts, and they have been widely employed as ORR catalysts in the past. The possible applications for nitride-based ORR catalysts are still unknown and have not been discussed in detail previously. 

In 2011, He et al. investigated the properties of a TiN/C composite during the ORR in proton-exchange membrane fuel cells [32]. Commercially-available TiN has also been employed as an effective cathode in LOBs [33] and reported elsewhere. They investigated in detail the electrochemical behavior of TiN as a cathode in LOBs using polarization curves, galvanostatic measurements, and alternative current (AC) electrochemical impedance spectroscopy (EIS). Origination of high cathodic current from the ORR was noticed at an onset potential of 3.8 V vs. Li/Li^+^, which was almost equal to the onset potential of noble metal Pt (4.0 V). Here, at a current of 0.5 mA and under a hybrid electrolyte medium with a weak acid solution and an organic electrolyte, galvanostatic measurements were carried out. The discharge curve obtained from the galvanostatic measurements exhibited a voltage plateau at 2.85 V and a capacity of 20 A h g^−1^. TiN catalyst as a cathode attained a power density of 1370 W kg^−1^ at 6 mA cm^−2^ [90]. The AC EIS measurements revealed an increase in the grain boundary after 200 h of discharging due to TiN electrode’s side reaction [91]. Thus, when compared with the electrochemical properties of noble metal electrodes in LOBs, the TiN electrode has significant drawbacks. 

More research has been conducted on TiN cathodes to overcome these disadvantages because they are much less expensive than noble metals when used as a cathode electrode in LOBs. Yarongwang et al. investigated nano as well as micro-sized TiN particles as the electrocatalyst for the ORR in LOBs with hybrid electrolytes [92]. Furthermore, in alkaline media, the ORR activity of these particles and corresponding pathways were also investigated Levich experiments in a rotating disk electrode (RDE). The ORR for the TiN particles were carried out using RDE cyclic voltammetry (CV) and RDE linear sweep voltammetry (LSV). The ORR in an aqueous solution was predicted to occur either through the 4e^−^ or the series 2e^−^ reduction pathways. In the four-electron reduction pathway, O_2_ is converted to OH^−^ through reduction by accepting 4e^−^ (1) and, in the 2e^−^ pathway, O_2_ is reduced to HO_2_^−^ followed by further reduction of HO_2_^−^ and then OH- by means of the following reactions (2)–(4), whereas SHE refers standard hydrogen electrode:

(1) $$2H_{2}O+O_{2}+4e^{-}\to 4\;OH^{-},\;0.401\;V\;vs.\;SHE$$

(2) $$H_{2}O+O_{2}+2e^{-}\to OH^{-}+O_{2}^{-}H,\;-0.065\;V\;vs.SHE$$

(3) $$HO_{2}^{-}+2e^{-}+H_{2}O\;\to 3OH^{-},\;0.876\;vs.\;SHE$$

(4) $$2HO_{2}^{-}\to O_{2}+2HO^{-}$$

**Figure 7:**
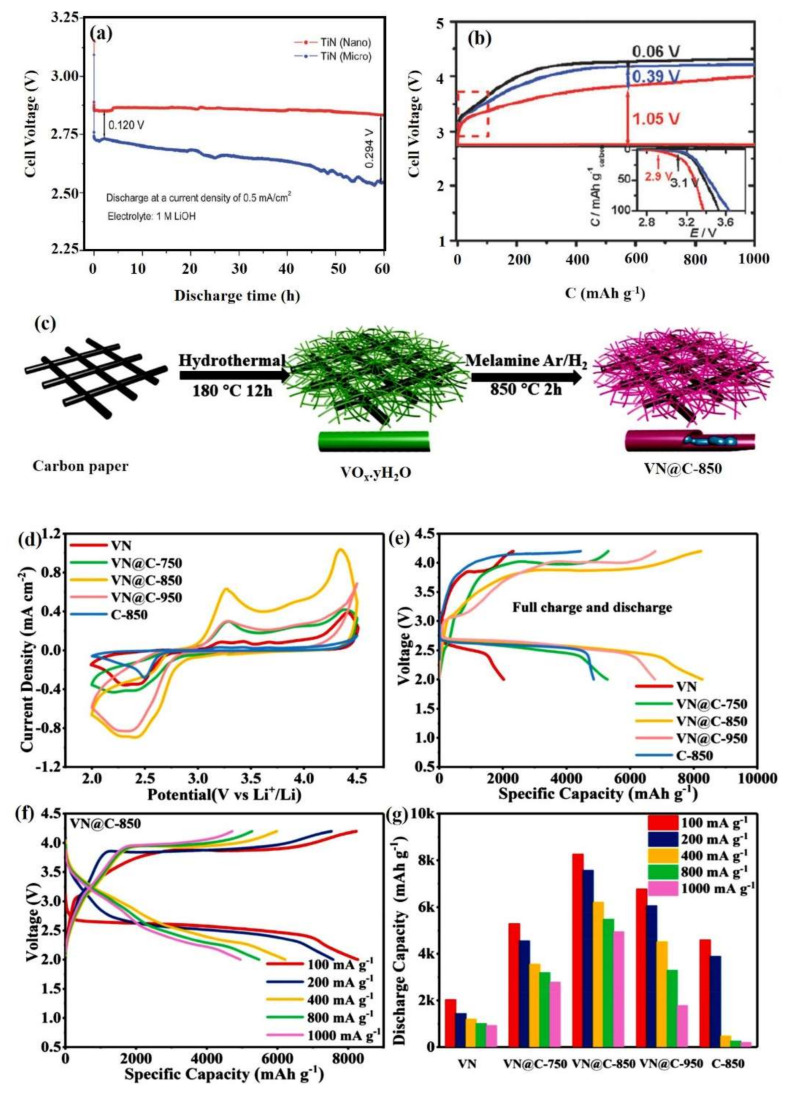
(**a**) Discharge plot for an LOB using TiN particles as a cathode catalyst [89]. Copyright 2012 The Royal Society of Chemistry. (**b**) Discharge plots for different TiN-based catalysts (m-TiN/VC and n-TiN/VC) and pristine VC (inset) @ 50 mA g_carbon_^−1^ [93]. Copyright 2013 The Royal Society of Chemistry. (**c**) Graphical illustration of the fabrication method for a VN@C-850 catalyst. (**d**) CV curves for VN, C-850, and VN@C at various calcination temperatures at 0.3 mV s^−1^ in the voltage range between 2.0 and 4.5 V. (**e**) Corresponding GCD plots (**f**) GCD curves for VN@C-850 at various current densities. (**g**) Discharge specific capacity of the cathode catalysts at various discharge rates [102]. Copyright 2020 Elsevier.

Here, the Levich experiments predicted that micro-sized TiN would follow the two-electron pathway, while TiN follows a dual path, in which two serial 2e^−^ steps occur at shorter intervals and produce comprehensive varied appearance due to the presence of parallel and serial 2e^−^ steps. The electrocatalytic activities of both nano- and micro-sized TiN particles for the ORR in LOBs were compared with nano-sized Mn_3_O_4_. Using the micro-sized TiN particles as a catalyst produced an initial discharge voltage of 2.74 V that decreased with time due to the presence of a small number of catalytic sites. It is also predictable that running the ORR through the 2e^−^ pathway would cause the piling up of electrons on the cathode surface, thus reducing the voltage gradually. In contrast, the nano-sized TiN without significant loss over time, had an even discharge voltage plateau around 2.85 V, and the observed catalytic voltage was also found to be slightly greater than the potential of Mn_3_O_4_ (2.80 V). The discharge curves of both types of NP are shown in Figure 7a [92].

Li et al. prepared micro- and nano-sized TiN particles on carbon Vulcan XC-72 (m-TiN/VC and n-TiN/VC) as a cathode for LOBs, following the work done by Yarongwang et al. [93] using a non-carbonate electrolytes. The template method was used to prepare Vulcan XC-72 carbon-supported nano-sized TiN [94]. LOBs with n-TiN, VC, and m-TiN as cathode catalysts were discharged and recharged at 50 mA g^−1^ (Figure 7b) [93]. Of the investigated cathode catalysts, n-TiN/VC was observed to have the lowest recharge voltage as compared to other prepared catalysts, with a potential gap of 1.05 V. The n-TiN/VC catalyst had the lowest onset potential for the OER from Li_2_O_2_ and the maximum reduction current for the ORR. Hence, the n-TiN/VC represents a suitable alternative for carbon materials, though further research is required to determine its dependency on electrolyte stability.

#### 3.3.2. Vanadium Nitride

Vanadium nitride (VN), an early TMN with a polar structure, is a promising material for use in energy storage devices due to its high structural stability, good electrical conductivity, and strong electron affinity [95,96,97,98]. Various VN nanostructures, such as nanoribbons [99], nanowires [100], and NPs [101], have been developed and employed as a potential host in lithium–sulfur batteries due to their strong affinity for the adsorption of polysulfides. As with other TMN materials, VN also readily undergoes electrochemical oxidation when used in an LOB; thus, there have been very few reports to date on VN-based catalysts for LOBs. 

To improve the stability of VN-based cathodes, recently, Sun et al. [102] proposed a strategy to introduce a carbon-coating layer over the nitride material. They prepared VN-coated N-doped carbon nanoribbons full-grown in situ over a carbon paper (VN@C) substrate (Figure 7c). The pyrolysis of the cathodes was conducted at temperatures of 750, 850, and 950 °C. As shown in Figure 7(d), the VN@C-850 cathode offered greater electrochemical activity by increasing the anodic and cathodic peak current densities in the formation and decomposition of Li_2_O_2_. From the charge–discharge plateau (Figure 7e–g), it was clearly confirmed that the VN@C-850 delivered the highest discharge capacity (8269 mAh g^−1^) at 100 mAcm^−2^ in a potential window of 2.0 to 4.2 V due to its greater OER/ORR catalytic activity during battery operation. These observations suggest that VN@C-850 could be a cutting-edge cathode for use in LOBs.

### 3.4. 2D Layered Transition Metal Carbides and Nitrides (MXenes)

This section focuses on the application of 2D TMC and TMN (MXene)-based materials in LOBs. Numerous review articles have dealt with the synthetic strategies and properties of MXenes [24,103,104,105,106], thus, we focus on summarizing current progress covering topics such as the role of 2D layered MXenes as essential electrodes in rechargeable LOBs. To date, the most common MXene-based materials used as potential electrodes in LOBs are titanium carbide (Ti_3_C_2_T_x_) and molybdenum carbide (Mo_2_C); these are explained in detail later in the section.

**Figure 8:**
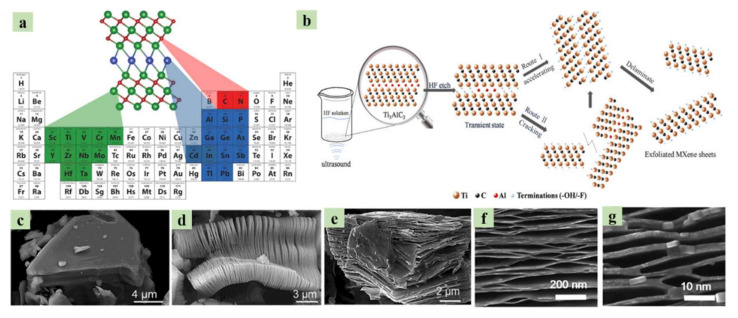
(**a**) M_n+1_AX_n_-producing elements from the MAX phase [114]. Copyright 2019 Elsevier. (**b**) Schematic diagram of the removal of A from MAX (exfoliation) and the conversion to MXenes [117]. Copyright 2018 Elsevier. (**c**–**e**) Scanning-electron microscopy (SEM) micrographs of exfoliated MXenes at different magnifications [116]. Copyright 2019 The American Chemical Society. (**f**,**g**) SEM micrographs of MXenes at different magnifications after delamination using various solvents [23]. Copyright 2017 The Royal Society of Chemistry.

In brief, MXenes have the chemical formula M*_n_*_+1_X*_n_*T*_x_*, in which M stands for a transition metal element (Ti, V, Mo, Nb, Cr, etc.) and X denotes the carbide or nitride. As illustrated in Figure 8a, they are atomically thin sheets of metal carbides, metal nitrides, or metal carbonitrides [24,107,108,109,110,111,112,113] with a surface functional group (T) of O^2−^, OH^−^, or F^−^ [114]. The first 2D MXene materials were developed by Gogotski’s research group at Drexel University in 2011. They derived 2D MXene from the MAX phase by removing the A group using etching [115], which is unlikely to block the transport of mobile ions [18]. These MAX phases consist of layers of TMCs or TMNs (M*_n_*_+1_X*_n_*) that are infused with layers of A-element atoms (i.e., Group 13 and 14 elements in the periodic table) [24]. After etching the surface of the MXene, it terminated with OH^−^ and F^−^ groups, which play a dominant role in deciding the characteristics of MXene compounds. These MX phases, which consist of several stacked layers of flakes or sheets with weak interlayer interactions, are subjected to delamination using solvents such as DMSO, DMF, and ethanol/water through ultra-sonication. Due to the presence of the surface-reacting X group, MXenes offer greater thermodynamic stability compared to their pure counterparts. Figure 8b presents a schematic representation of the process of producing an MXene from a MAX phase [116,117]. To date, over 70 MAX phases and over 20 MXenes have been identified. The ultra-sonication time and the choice of surfactant intercalation agent strongly affect the morphology of MXene compounds, which can include wrinkled sheets, spheres, nanoplates, and scrolls. An example of MXene sheet morphology is shown in the SEM micrographs in Figure 8c–g [23,116]. In contrast to graphene materials, MXenes offer a variety of chemical compositions due to the atomic layer thickness of the TMCs and TMNs. 

#### 3.4.1. 2D Layered Titanium Carbide (Ti_3_C_2_T_x_)

While porous carbonaceous materials are highly useful for enhancing ORR activity, their OER activity remains low, leading to a high charging potential of over 4.5 V and the decomposition of the electrolyte, thus weakening the performance of LOBs. For this reason, noble metal catalysts such as Pt, Au, and RuO_2_ have been employed in LOBs, but their high cost and poor specific capacity have restricted their use in commercial applications. Recently, 2D metal carbides have gained attention because of their advantageous properties, including the presence of several valence states, low resistivity, stronger electrochemical properties, and low cost. Due to the presence of reactive functional groups such as conductive metallic carbides and terminal transition metal atoms (Ti) on their surface, Ti_3_C_2_T_x_ MXenes are considered a promising candidate for various energy storage devices, including LIBs, sodium ion batteries, Li-S batteries, supercapacitors, and LOBs. They also have a specific surface morphology with ultra-high conductivity and malleable interlayer spaces. For these reasons, the lithium diffusion barrier of Ti_3_C_2_T_x_ MXenes (0.07 eV) is lower than that of commercial graphite electrodes (0.3 eV) due to the higher number of active sites, which in turn favors the rapid migration of lithium ions and enhanced charge–discharge characteristics. Recently, due to its high conductivity and hydrophilicity, Xue et al. [118] reported excellent ORR behavior with outstanding electrochemical activity for an Mn_3_O_4_-Ti_3_C_2_ catalyst, while Zou et al. [119] fabricated an MOF-derived Ni-Co mixed sulfide on a Ti_3_C_2_ matrix and used it as an active catalyst for OER reactions in zinc–air batteries. Based on their exceptional ORR/OER catalytic properties, Ti_3_C_2_T_x_ MXenes with different active components such as TiO_2_ [120], MoS_2_ [121], SnS [122], Fe_3_O_4_ [123], g-C_3_N_4_ [124], ZnO [125], and MnO_2_ [126] have been widely employed as cathodes in LIBs, SCs, and SIBs. However, Ti_3_C_2_T_x_ MXenes have rarely been reported as a cathode material for LOBs. Hence, this is an emerging topic that requires further attention. Some interesting composite cathodes based on Ti_3_C_2_T_x_ MXenes for LOBs are discussed below. 

Initially, Zheng et al. [127] investigated the reaction kinetics for the OER and ORR in LOBs using oxygen-enriched TiO_2_ NPs on Ti_3_C_2_T_x_ (V-TiO_2_/ Ti_3_C_2_T_x_) as a potential oxygen electrode. This oxygen electrode, processed using ethanol thermal treatment, exhibited excellent catalytic activity and high battery performance, with a maximum specific capacity of 11,487 mAh g^−1^, an overpotential of 0.21 V, and high durability even after 200 cycles. 

**Figure 9:**
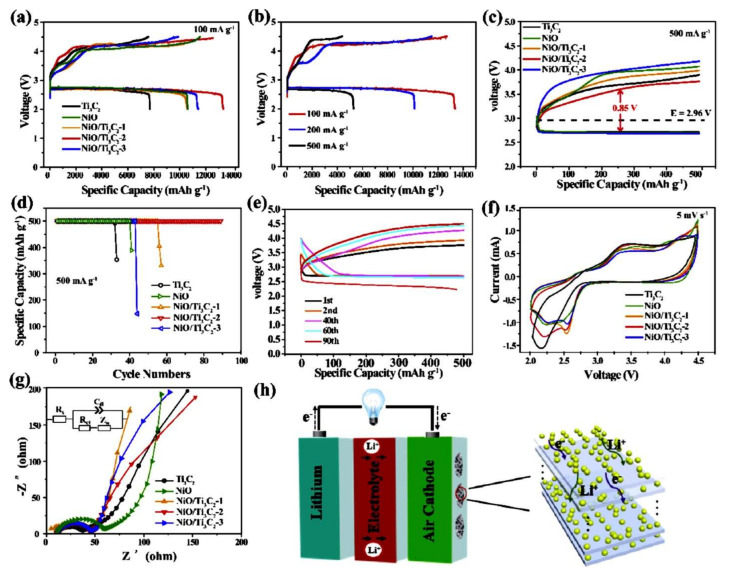
(**a**) GCD profile of an LOB using NiO-based Ti_3_C_2_ catalysts @ 100 mA g^−1^. (**b**) GCD of NiO-Ti_3_C_2_-2 at various current densities. (**c**) GCD profile of an LOB using NiO-based Ti_3_C_2_ catalysts @ 500 mA g^−1^. (**d**) Cycling behavior of NiO-based Ti_3_C_2_ catalysts. (**e**) GCD plot of NiO-Ti_3_C_2_-2 at a fixed current rate of 500 mA g^−1^. (**f**,**g**) CV and electrochemical impedance analysis (EIS) plots of NiO-based Ti_3_C_2_ catalysts. (**h**) Graphical representation of charge–discharge mechanisms in an LOB [128]. Copyright 2020 The American Chemical Society.

Over the past decade, Ni-based oxide materials have been increasingly incorporated into renewable storage devices due to their high catalytic activity and general abundance and availability. However, their poor intrinsic electrical conductivity and tendency to agglomerate have limited their commercial viability. To overcome these shortcomings, 2D layered materials such as graphene and reduced graphene oxides have been employed. Recently, NiO-Ti_3_C_2_T_x_ MXene catalysts were proposed as a low-cost electrocatalyst for LOBs by Li et al. [128]. Different combinations of ultrasonically processed NiO-Ti_3_C_2_T_x_ MXene electrodes displayed excellent electrochemical behavior, with a superior initial discharge capacity of 13,350 mAh g^−1^ @ 100 mA g^−1^ (Figure 9a). However, as shown in Figure 9b, the capacity drastically fell to 5790 mAh g^−1^ @ 500 mA g^−1^. Furthermore, it can be seen in Figure 9c–e that the capacity of the electrodes depended on the NiO loading mass, rising initially before subsequently declining. The NiO-Ti_3_C_2_-2 electrode exhibited a minimum charge and discharge overpotential of 0.64 and 0.21 V, respectively. The same electrode offered an excellent capacity retention of over 90 cycles with the highest round-trip efficiency of 76.4%. This electrocatalytic activity was further supported by CV and EIS analyses (Figure 9f,g), confirming the rapid transport of lithium ions in LOBs (Figure 9h). In a similar manner, CoO-incorporated Ti_3_C_2_T_x_ MXene nanosheets were recently synthesized using a hydrothermal process, producing excellent lithium oxygen storage performance with the highest initial cycle capacity at 16,220 mAh g^−1^ and an extended life, of over 160 cycles [129].

#### 3.4.2. 2D Layered Molybdenum Carbides (Mo_2_CT_x_)

In the recent years, numerous reports have been published on the use of Mo_2_C nanocatalysts as cathodes to reduce the charge overpotential in LOBs due to advantages such as the variety of valence states, high electrical conductivity, stronger electrochemical activity, and abundance. These compounds also have a Pt-like structure and thus represent a cost-effective alternative for noble metals. Recently, Yu et al. [130] prepared Mo_x_C (α-MoC_1−x_ and β-Mo_2_C) porous nanorods derived from Mo-MOFs and employed them as the cathode in an LOB. Of the two as-prepared cathodes, α-MoC_1−x_ exhibited improved catalytic properties due to a low charge transfer resistance of 395.8 Ω and a strong O_2_ absorbability of −1.87 eV. The maximum discharge capacity of α-MoC_1−x_ was found to be 20,212 mAh g^−1^ with a discharge potential of 2.62 V. The prepared cathode also exhibited long-term cycling stability over 100 cycles with a high round-trip efficiency of 70%. 

Of the various forms of molybdenum carbide, Mo_2_C is the most stable because of its in-built OER and high conductibility. Based on these characteristics, a few studies have focused on developing Mo_2_C-based composite cathodes. For instance, an Mo_2_CT_x_/CNT composite in non-aqueous LOBs has been reported to deliver the highest discharge capacity of 9100 mAh g_CNT_^−1^ at a cut-off potential of 2.0 V, which is relatively high compared to a pristine CNT electrode (5950 mAh g_CNT_^−1^) [131]. This suggests that the combination of Mo_2_CT_x_ with CNTs facilitated the transport of Li_2_O_2_, thus enhancing the overpotential, capacity, and shelf life of the electrode. In a similar manner, Mo_2_C NPs on CNFs (MCNFs) fabricated using electrospinning delivered a maximum specific capacity of 10,509 mAh g^−1^ at a current density of 100 mA cm^−2^ [132]. The increase in capacity compared to Mo_2_CT_x_/CNTs can be attributed to the high diffusion flux of lithium ions and O_2_, which prevents the blockage of Li_2_O_2_ in the binder-free structure of MCNFs. In addition, the rich active sites and rapid electronic migration in MCNFs reduced the charge–discharge potential gap and led to a stable capacity of 500 mAh g^−1^ even after 124 cycles.

**Figure 10:**
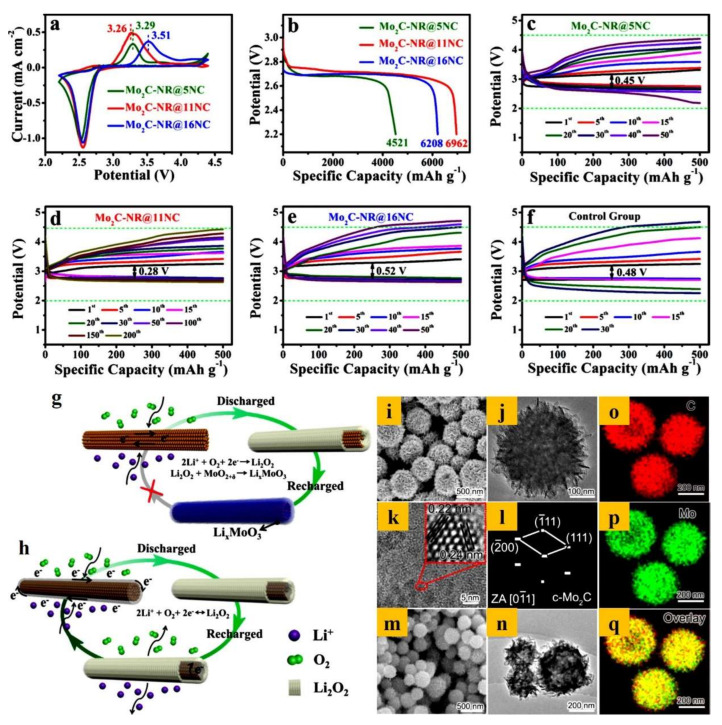
(**a**) CV plot of NC-doped Mo_2_C-NR (Mo_2_C-NR@xNC; x = 5, 11, and 16%) for an LOB under an O_2_ atmosphere at 0.1 mVs^−1^. (**b**) GCD plot of Mo_2_C-NR@xNC (x = 5, 11, and 16%) at 100 mA g^−1^. (**c**) Discharge profile for an LOB with Mo_2_C-NR@5%NC. (**d**) Discharge profile of an LOB with Mo_2_C-NR@11%NC. (**e**) Discharge profile of an LOB with Mo_2_C-NR@16%NC. (**f**) Discharge plot of the control group at 100 mA g^−1^. (**g**,**h**) Graphical representation of an LOB cell with Mo_2_C-NR and Mo_2_C-NR@11 NC catalysts [133]. Copyright 2018 The American Chemical Society. (**i**) FE-SEM image of Mo_2_C-C. (**j**,**k**) HR-TEM image of Mo_2_C-C and (**l**) the corresponding FFT pattern. (**m**,**n**) FE-SEM and HR-TEM images of the control sample carbon. (**o**–**q**) EDS mapping of C and Mo in Mo_2_C-C [134]. Copyright 2020 Elsevier.

A binder/current-collector-free 3D foam consisting of Mo_2_C nanorods with different concentrations of nitrogen-doped carbon (Mo_2_C-NR@NC) was prepared by Sun et al. and directly employed as an O_2_ electrode [133]. During the charge–discharge process, the as-prepared electrode minimized the parasitic reactions in LOBs and enhanced the energy density and specific capacity. The Mo_2_C-NR with 11% NC (Mo_2_C-NR@11NC) exhibited the highest activity for OER and ORR catalytic processes. As shown in Figure 10a, the cell operated in a voltage window of 2.2 to 4.4 V. The excellent capacity of the Mo_2_C-NR@11NC electrode is illustrated in Figure 10b, with a maximum discharge capacity of 6962 mAh g^−1^ vs Li^+^/Li. The low charge–discharge potential gap was also assessed by subjecting electrodes with different concentrations of NC to cycling at a constant current density rate of 100 mA g^−1^. Figure 10c–f shows that the cell with the Mo_2_C-NR@11NC electrode offered the lowest potential gap (0.28 V), which may be due to the avoidance of parasitic reactions in LOBs. The mechanisms involved in the electrochemical process in an LOB were also proposed (Figure 10g). In brief, Mo_2_C-NR@11NC reduced the generation of MoO_2_ on the Mo_2_C surface, while the other two electrodes generated significant amounts of MoO_2_ on the surface, which readily underwent a reaction with Li_2_O_2_, leading to the formation of Li_x_MoO_3_ which dissolves in the electrolyte and limits the reversibility of the electrode. 

Recently, Mo_2_C/C with a porous nanoflower hierarchical architecture was used to produce carbon nanosheets decorated with Mo_2_C quantum dots [134]. Figure 10i–q presents images of ultra-thin 2D nanosheets with Mo_2_C that has self-assembled into a nanoflower morphology with an average diameter of 200 to 500 nm. The porous nanoflower morphology facilitated the migration of Li^+^ and accommodated Li_2_O_2_, thus exhibiting an ultra-high capacity (7500 mAh g^−1^) and superior cycling stability (104 cycles). The passivation of the positive electrode by Li_2_O_2_ and Li_2_CO_3_ was evaluated theoretically using an MoC hollow sphere electrode by Zakharchenko et al. to improve the performance of LOBs [135].

Previous reports on CNT-based Mo_2_C cathodes have highlighted promising results in terms of improving LOB performance. Oh et al. prepared bifunctional, centipede-like MoC-Mo_2_C on an N-doped CNT cathode for high-performance LOBs [136]. Interestingly, the prepared cathode had a nanorod morphology with excellent catalytic activity, which in turn facilitated electron transport through the N-CNT and provided sufficient space to accommodate Li_2_O_2_. The Mo_2_C-MoC/NCNT nanorods acted as a promising candidate for use in LOBs due to their high discharge capacity (34,862 mAh g^−1^) and long-term stability. A composite cathode prepared by growing CNTs and carbon-wrapped Mo2C NPs directly on Ni foam was employed as a noble catalyst for LOBs, exhibiting outstanding cycling performance with a capacity of 10,400 mAh g^−1^, a charge cut-off potential of 4 V, and a low charge–discharge voltage gap of 0.9 V [137].

Another important form of Mo_2_C that has attracted recent attention is MoO_2_-MoC cathodes. Lu et al. designed a heterostructured bioinspired Mo_2_C-MoO_2_/N-doped carbon foam catalyst derived from an ELKA 16-FLAG precursor [138]. The reported catalyst exhibited an outstanding overall rate performance with excellent cyclability. This catalytic performance can be ascribed to the specific hierarchical microporous structure of the 3D carbon foam. In addition, the doping of nitrogen into the carbon foam enhanced the conductivity and catalytic activity. Similar research was carried out by Wu et al., who fabricated a bifunctional Mo_2_C-MoO_2_/rGO catalyst and examined the synergistic effect of rGO and Mo_2_C-MoO_2_ heterostructures on the performance of batteries [139]. Though the charging voltage (4.5 V) of the catalyst was high, it did not decompose the carbon matrix (rGO) and produced a high round-trip efficiency of 89%. The capacity of Mo_2_C-MoO_2_/rGO was approximately 2365 mAh g^−1^, indicating that the rationally designed catalyst had higher absorbability. 

#### 3.4.3. 2D Layered Transition Metal Nitrides

In general, 2D layered TMNs are highly electronically conductive compared to their 2D layered TMC counterparts and suitable for the majority of electronic storage devices. However, its practical applications are largely hindered by their complicated preparation process. According to earlier report by Shein et al., the threshold energy required for the conversion of M_n+1_N_n_ is comparatively higher than that of Mn+1Cn from their corresponding MAX (M_n+1_AlN_n_ and M_n+1_AlC_n_) phase [140]. It is important to notice that acidic solutions are not opt to remove the ‘A’ phase (Al, Sn) from MAX for 2D layered TMNs due to its less cohesive energy, which leads to make them unstable when it dissolved in HF solution and failed completely. Hence, the synthesis of 2D layered nitride based MXenes (2D TMNs) are highly complex and cost-effective [141,142]. Owing to these factors, only a few 2D layered TMNs have been reported. Urbankowski et al. have successfully derived the Ti_4_N_3_ MXene from Ti_4_AlN_3_ using molten fluoride salt as etchant at 550 °C [143]. Ti_2_N based MXenes have been successfully exfoliated by Bhuvaneswari et al. using KF and HCl as the selective etchants [144]. Additionally, an ammonization of TMCs to convert into TMNs have also reported recently [145]. Furthermore, a scalable-salt templating processed 2D molybdenum, tungsten, and vanadium nitride films have prepared and studied experimentally and computationally by Xiao et al. [146]. 

Besides complex synthesis process, these TMNs possess outstanding mechanical and electrical properties and better chemical and thermal stabilities, and finds application in diverge fields such as storage and conversion devices, sensors, electronics, fuel cells, and catalysis [141]. However, the reports on 2D layered TMNs as cathode catalysts for LOB are very limited. For instance, as discussed earlier (Section 3.3.2), Sun et al. have fabricated N-doped carbon incorporated vanadium nitride (VN) cathode catalysts with significantly enriched catalytic activity for OER-ORR processes for LOBs. Because of their high nitrogen content and appreciable electric conductivity, the prepared catalyst assembled LOB cells have delivered the outstanding capacity of 8269 mAh g^−1^ with a GCD voltage gap of 0.88 V [102]. On the other hand, the 2D carbonitride based FeCo-N-C catalyst have been considered as a potential cathode material for LOBs because of its highly active sites. Interestingly, this microporous Fe, Co doped catalyst has offered durable cycling stability up to 75 cycles with the maximum discharge capacity as high as 17,200 mAh g^−1^ at a low current rate [147]. Shukla et al. recently further evaluated sulfide functionalized vanadium and titanium based cathode catalysts include V_2_NS_2_ and TiN_2_S_2_ for energy storage devices through the first principle DFT methodology [148]. Zhao et al. have obtained a very good electrocatalytic activity as well as long-term durability while using Pt supported graphitic C_3_N_4_ catalysts for LOBs [149]. Although 2D layered TMNs are capable to offer excellent reaction sites for OER/ORR processes, thereby leading to provide high specific capacity, it is hard to decompose their bi-products (lithium peroxide) and complex experimental processes restrict them from commercialization.

Table 1 summarizes the electrochemical and storage performance of various TMC- and TMN-based cathode electrocatalysts for LOBs applications.

## 4. Summary, Outlook, and Future Prospects

In summary, LOBs have strong potential as an energy source for future electronic devices and hybrid plug-in vehicles due to their higher energy density. In order to expand the commercial market for LOBs, a significant volume of research has been conducted over the past decade. However, the commercial development of LOBs has faced practical hurdles such as their short lifespan, poor specific capacity, poor polarization during charging and discharging, and slow kinetics of the electrochemical mechanisms for OER/ORR catalytic activity. In order to overcome these issues, a variety of cathode catalysts, including carbonaceous materials, noble metal/transition metal oxides, metal chalcogenides/phosphides, MOFs, TMCs, and TMNs have been studied. Of these materials, TMCs and TMNs have exhibited particular promise due to their specific composition and unique physicochemical properties.

In this focused review, we have summarized the electrochemical catalytic behavior and storage performance of both bulk and 2D layered TMCs and TMNs for use in LOBs. Compared to bulk TMCs and TMNs, 2D MXenes and their composites offer outstanding capacity and a high round-trip efficiency. Recently, research has focused on extending the range of MXenes by exploring different combinations of elements to improve the performance of LOBs. However, their commercial usefulness is limited by their high cost (e.g., ~$366 per 100 g of powder, Ukraine Y-Carbon Ltd., excluding exfoliation, delamination, and other purification processes) and structural variability. Therefore, cost-effective fabrication processes need to be developed to reduce the cost of precursors and final products, while suitable solvents that can ensure the structural stability of MXenes for use in energy storage devices need to be identified.

In this regard, the source in attaining cathode catalysts’ must be expanded. To reduce the capital cost as well as apprehend a green and sustainable LOB electrocatalytic mechanism, the catalysts should be anticipated to derive from daily waste products. The side reactions and their corresponding by-products should be minimized during electrochemical process by the way of designing a new type of catalyst materials and to apply different synthesis strategies. In order to enhance the safety features of the LOB, the volatile liquid LiPF_6_ electrolyte should be substituted by solid and quasi-solid state electrolytes. Henceforth, more attention will be paid for the interface reaction at the electrode-electrolyte surface, protection of anode from passivation, and conductivity of the electrolyte.

In addition, CO_2_ and H_2_O contamination and the high discharge current rate in Li–O_2_ electrochemistry limit the formation of Li_2_O_2_, leading to poor cycling stability and cell capacity. To avoid these problems, the repeatability and reproducibility of Li–O_2_ cells need to be guaranteed by ensuring sealing of the cell hardware, thus supporting the catalytic mechanisms during the electrochemical reactions. Overall, this review article expands the understanding of OER/ORR catalytic mechanisms and provides guidelines for future research on TMC- and TMN-based cathode catalysts, which can be used to significantly advance LOB technology.

## Figures and Tables

**Table 1:** Electrochemical and storage performance of TMC- and TMN-based cathode electrocatalysts for LOBs.

Materials	Morphology	CD-Potential Gap (V)	Capacity (mAh g^−1^)/Current Density (mA g^−1^)	Capacity Retention	Ref
TiC	Nanosheets	1 V	500 at 1 mA cm^−2^	98% after 100 cycles	[67]
Ru-TiC	Nanowire	0.91 V	1.6 mAh cm^−2^ at 0.1 mA cm^−2^	~61% after 270 cycles	[74]
TiC-cloth	Nanowire	0.6 V	0.5 mAh cm^−2^ at 0.1 mA cm^−2^	~92% after 100 cycles	[75]
graphene@Fe/Fe_3_C	Three-dimensional porous structure	0.61 V	7150 at 0.1 mA cm^−2^	n.a	[83]
F@NG-NCNT	Bamboo tubular	1.1 V	6966 at 0.1 mA cm^−2^	100% after 30 cycles	[84]
Fe/Fe_3_C–CNFs	Nanofibers	1.05 V	6920/80	n.a	[85]
(V-TiO_2_/Ti_3_C_2_T_x_)	Nanosheet	0.21 V	11,487/100	79% after 200 cycles	[127]
NiO/Ti_3_C_2_	Accordion nanosheets	1.07 V	13,350/100	Maintains stable capacity after 90 cycles	[128]
CoO/Ti_3_C_2_T*_x_*	Layered nanosheet	1.02 V	16,220/100	n.a	[129]
Carbon-wrapped Mo_2_C/Ni-foam	Nanoparticles	0.9 V	10,400/100	Maintains stable capacity after 200 cycles	[137]
Mo_2_C/C	Nanoflowers	1.2 V	7500/100	Maintains stable capacity after 104 cycles	[134]
Mo_2_C/CNF	Nanoparticles	1.0 V	10,509/100	Maintains stable capacity after 124 cycles	[132]
Mo_2_CT_x_ MXene/CNT	Nanoporous	2.0 V	5950/100	Maintains stable capacity after 40 cycles	[131]
Mo_2_C-NR@NC	Nanorods	0.28 V	6962/100	n.a	[133]
Mo_2_C-MoC	House penticide	n.a	34,862/200	Maintains stable capacity after 162 cycles	[136]
MoO_2_/Mo_2_C	Porous nanocrystals	0.21 V	5000/1000	Maintains stable capacity after 40 cycles	[138]
Mo_2_C-MoO_2_	Porous nanosheet	0.56 V	2365/200	Maintains stable capacity after 100 cycles	[139]
n-TiN/VC	Nanoparticles	0.39 V	6407/50	n.a	[90]
VN @C	nanoribbon	0.88 V	1000/100	Maintains stable capacity after 183 cycles	[102]
Fe, Co–co-doped C-N	polyhedra	1.0 V	800/500	Maintains stable capacity after 56 cycles	[147]
Pt supported g-C_3_N_4_	nanosheet	1.5 V	17,059.5/100	Maintains stable capacity after 100 cycles	[149]
